# Efficacy and safety of hepcidin-based screen-and-treat approaches using two different doses versus a standard universal approach of iron supplementation in young children in rural Gambia: a double-blind randomised controlled trial

**DOI:** 10.1186/s12887-016-0689-4

**Published:** 2016-09-01

**Authors:** Rita Wegmüller, Amat Bah, Lindsay Kendall, Morgan M. Goheen, Sarah Mulwa, Carla Cerami, Diego Moretti, Andrew M. Prentice

**Affiliations:** 1 MRC Unit The Gambia/MRC International Nutrition Group, Keneba, The Gambia; 2 MRC Unit The Gambia, Fajara, The Gambia; 3 Department of Microbiology and Immunology, University of North Carolina, Chapel Hill, NC 27599 USA; 4 Department of Epidemiology, Gillings School of Global Public Health, University of North Carolina, Chapel Hill, NC 27599 USA; 5 Laboratory of Human Nutrition, Institute of Food, Nutrition and Health, ETH Zurich, 8092 Zurich, Switzerland

**Keywords:** Hepcidin, Iron supplementation, Iron deficiency, Iron deficiency anaemia, Anaemia, Safety, Children, Gambia, Sub-Saharan Africa

## Abstract

**Background:**

Iron deficiency prevalence rates frequently exceed 50 % in young children in low-income countries. The World Health Organization (WHO) recommended universal supplementation of young children where anaemia rates are >40 %. However, large randomized trials have revealed that provision of iron to young children caused serious adverse effects because iron powerfully promotes microbial growth. Hepcidin – the master regulator of iron metabolism that integrates signals of infection and iron deficiency – offers the possibility of new solutions to diagnose and combat global iron deficiency. We aim to evaluate a hepcidin-screening-based iron supplementation intervention using hepcidin cut-offs designed to indicate that an individual requires iron, is safe to receive it and will absorb it.

**Methods:**

The study is a proof-of-concept, three-arm, double blind, randomised controlled, prospective, parallel-group non-inferiority trial. Children will be randomised to receive, for a duration of 12 weeks, one of three multiple micronutrient powders (MNP) containing: A) 12 mg iron daily; B) 12 mg or 0 mg iron daily based on a weekly hepcidin screening indicating if iron can be given for the next seven days or not; C) 6 mg or 0 mg iron daily based on a weekly hepcidin screening indicating if iron can be given for the next seven days or not. The inclusion criteria are age 6-23 months, haemoglobin (Hb) concentration between 7 and 11 g/dL, z-scores for Height-for-Age, Weight-for-Age and Weight-for-Height > -3 SD and free of malaria. Hb concentration at 12 weeks will be used to test whether the screen-and-treat approaches are non-inferior to universal supplementation. Safety will be assessed using caregiver reports of infections, in vitro bacterial and *P. falciparum* growth assays and by determining the changes in the gut microbiota during the study period.

**Discussion:**

A screen-and-treat approach using hepcidin has the potential to make iron administration safer in areas with widespread infections. If this proof-of-concept study shows promising results the development of a point-of-care diagnostic test will be the next step.

**Trial registration:**

ISRCTN07210906, 07/16/2014

## Background

### Iron deficiency: global prevalence, health consequences, and barriers to progress in elimination

Iron deficiency (ID), leading to iron deficiency anaemia (IDA) and impaired neurocognitive development, remains the most pervasive nutritional deficiency worldwide [1]. Using currently accepted criteria the prevalence rates for IDA in young children frequently exceed 50 % in low-income countries (as is the case in The Gambia) resulting in impaired immunocompetence and brain development and thus leading to substantial loss of human potential [2]. Low cost iron supplements are efficacious in combatting IDA; thus in countries with anaemia rates of >40 %, WHO recommended universal supplementation of pregnant women and young children. However, in 2006, the Pemba Trial was prematurely terminated due to significant increases in the number of serious adverse outcomes and deaths in young children receiving iron-folic acid supplements [3]. This result was attributed to a malign interaction between iron, folic acid and malaria (since a parallel trial in a non-malarious area of Nepal revealed no increase in adverse outcomes), and WHO revised its policy guidance for iron supplementation to a more cautionary approach in areas with endemic malaria [4]. The new guidelines recommended adoption of targeted supplementation (a screen-and-treat approach) or the use of centralized or point-of-use food fortification using micronutrient powders, which was considered likely to be a safer option. However, recent studies, including a very large trial from a non-malarious area in Pakistan [5], have revealed important evidence of medically significant adverse outcomes associated with iron administration. It is assumed that all of these adverse outcomes may be attributable to host-pathogen competition for iron whereby supplemental iron has favoured pathogens more rapidly than their host. Thus there is an urgent need: a) to understand the pathways by which provision of iron favours pathogen virulence; and b) to use this knowledge to design safer modes for preventative and therapeutic provision of iron to infants and young children living in infectious environments in order to reduce infections and to allow maximal brain development.

### Evidence of detrimental effects of iron

Iron is unique among nutrients in being both essential and highly toxic. This tension has driven the evolution of complex systems for regulating iron absorption, and safely chaperoning it during transportation, storage and utilization in a vast majority of living organisms including humans of complex systems for regulating iron absorption, and safely chaperoning it during transport, storage and utilization [6]. The discovery of the iron-regulatory hormone hepcidin has thrown a sharp new focus on the adaptive/protective value of maintaining strict physiological control over iron absorption.

Initially the Pemba results were viewed with some scepticism in many quarters and/or were assumed to only affect malarious regions, as well as to be confined to supplementation as opposed to fortification. Yet, additional intervention trials have shown that administration of iron, either alone or as part of multiple micronutrient formulations, can lead to serious adverse effects as follows: a) a second trial in Tanzania showed an association between micronutrient supplements containing iron and the risk of malaria [7]; b) two trials of iron-fortified foods given to children in Côte d’Ivoire [8] and Kenya [9] caused significant adverse re-profiling of the gut microflora and evidence of intestinal inflammation; c) a large cluster-randomized single blind trial of iron-containing Sprinkles in 17,000+ Pakistani children has revealed significant increases in diarrhoea and pneumonia rates and a very significant increase in severe and bloody diarrhoea [5]. Finally, a recently published trial in young Ghanaian children receiving multiple micronutrient powders showed a trend towards lower malaria rates in the group receiving iron but an increase in hospitalisations among the iron-receiving group [10].

### The role of hepcidin – the master regulator of iron metabolism

Iron homeostasis, and its distribution within the body, is maintained by regulating absorption of iron through duodenal enterocytes and by controlling the rate of iron recycling through macrophages. Hepcidin is a small peptide hormone that binds to and causes the degradation of ferroportin, an iron export protein highly expressed by enterocytes and macrophages [11]. High levels of hepcidin thereby inhibit absorption of dietary iron and lock iron in macrophages, rapidly depleting serum iron (causing the protective hypoferraemia of the acute phase response) and lowering iron availability for erythropoiesis [12]. An abundance of genetic evidence in humans and experimental animals indicates that the ferroportin-hepcidin interaction is the dominant and non-redundant regulator of iron balance and iron distribution. Regulation of hepcidin is complex – iron accumulation induces hepcidin, providing a negative feedback loop to maintain homeostasis, but hepcidin levels are also increased by inflammatory signals arising during infections. Iron deficiency and erythropoietic drive suppress hepcidin, releasing iron from cellular stores and increasing dietary iron absorption. Importantly, each of these signals is variable, and the balance between them determines hepcidin synthesis. Thus, an iron deficient individual may have high serum hepcidin due to an acute infection, but on the other hand severe anaemia may suppress hepcidin even in the presence of inflammation.

### Iron redistribution as a component of innate defence against infection

It is widely accepted that the hypoferraemia of the acute phase response represents a highly conserved component of innate defence against a broad-spectrum of extracellular organisms that could elicit a rapidly fatal septicaemia if allowed unrestricted access to circulating iron. Several studies have proposed that iron-requiring intracellular organisms might have evolved their niche specificity precisely to capitalize upon the consequent iron-rich environment in macrophages [13]. Such interactions may play a significant role in explaining susceptibility to secondary infections. Experimental validation in animal and human studies may have important therapeutic implications.

### Hepcidin-guided iron supplementation

Because hepcidin reports on the balance of iron status and inflammation, and because hepcidin also determines how well oral iron is absorbed [11], low hepcidin levels indicate both a requirement for iron and an ability to utilize it if provided. Individuals with high hepcidin may be iron replete, or inflamed, or both, but will not be able to absorb oral iron efficiently. In our recently published paper this is clearly illustrated in young children, and our results suggest a cut-off of 5.5 ng/mL [14]. As discussed above, iron supplementation may provide limited benefit and be associated with deleterious consequences when given in the presence of inflammation. Therefore, providing an extra decision point (do not give iron unless hepcidin is below a cut-off level) in iron supplementation programs should make them both safer and more efficient. This will permit safe iron supplementation of young children in infectious areas allowing them to reach their full human potential.

### Multiple micronutrient powders (MNP)

In this trial we will use a micronutrient powder (MixMe) as it is used by the United Nations Children’s Fund and the World Food Programme. In a Cochrane systematic review (comprising eight large trials from a variety of settings, including malaria-endemic areas) assessing the effects and safety of home fortification with multiple micronutrient powders of foods consumed by children under two years, anaemia was reduced by 31 % and iron deficiency by 51 % [15]. The review found no difference in the effect of the intervention among children living in malaria endemic areas or areas with sporadic malarial cases. Based on this review WHO guidelines on home fortification were developed, strongly recommending home fortification with micronutrient powders to improve iron status and reduce anaemia among infants and young children 6-23 months of age [16].

More recently two other trials investigating the effect of MNP, one in Ghana and one in Pakistan, have been published. The trial in Pakistan showed significant increases in diarrhoea and bloody diarrhoea and pneumonia rates [5], whereas the trial in Ghana showed a trend towards lower malaria rates in the group receiving iron but an increase in hospitalisations among the iron group [10]. Although our trial will not be powered to assess morbidity we will monitor our children very closely by collecting morbidity data twice weekly.

### Study objectives

The *primary objective* is to evaluate whether weekly hepcidin-based screen-and-treat at 12 mg iron/day, screen-and-treat at 6 mg iron/day and 12 mg iron/day universal supplementation for 12 weeks are all non-inferior. The primary endpoint is Hb (measured using a Medonic haematology analyser in the laboratory) at day 84. Secondary endpoints related to the primary objectives are:

i) Proportion of anaemia (Hb < 11 g/dL) at Day 84;
ii) Proportion of iron deficiency (sTfR/logFerritin ratio > 3.2 or > 2.0 in the presence of inflammation (CRP > 5 mg/L) and ferritin < 12 μg/L or < 30 μg/L in the presence of inflammation) at Day 84;
iii) Proportion of iron deficiency anaemia (Hb < 11 g/dL and sTfR/logFerritin ratio > 3.2 or > 2.0 in the presence of inflammation and ferritin < 12 μg/L or < 30 μg/L in the presence of inflammation) at Day 84;
iv) Ferritin (only participants without inflammation), soluble transferrin receptor (sTfR), transferrin and transferrin saturation (TSAT) at Day 84.

The *secondary objectives* are the evaluation of i) the feasibility of adopting a hepcidin-based screen-and-treat approach to iron supplementation in young children (number of weeks supplemented with iron); ii) beneficial effects with screen-and-treat with respect to maternal reporting of child illness and safety indices (inflammatory and immune activation markers, faecal gut inflammation markers, gut microbiota, *ex-vivo* bacterial and *P. falciparum* growth); and iii) overall iron absorption over the 12 week supplementation period in the three study arms (sub-study).

## Methods/design

This paper describes the methodology of a randomized controlled non-inferiority trial comparing three different arms. Two arms are screen-and-treat approaches using hepcidin to indicate whether the body is ready to absorb iron and thus making it safe to give iron and the third arm is universal iron supplementation (recommended by WHO in non-malarial areas). Children randomized to the universal arm will get 12 mg iron daily for 12 weeks and children randomized to the screen-and-treat groups will receive 12 mg or 6 mg iron daily if their weekly hepcidin indicates a low value (<5.5 ng/mL), or 0 mg iron if their hepcidin indicates a high value (≥5.5 ng/mL) for the next seven days for a total of 12 weeks.

### Study site

Study participants will be recruited from 12 communities in Jarra West (Soma, Karantaba, Kani Kunda, Sankwia, Mansakonko, Pakalinding, Jenoi and Si Kunda) and Kiang East (Toniataba, Jiffin, Kaiaf and Genieri), in the Lower River Region of The Gambia. The communities around the town of Soma are approximately 170 km east of the capital Banjul. The town and some of the surrounding villages have an unreliable electrical power supply. All communities have access to borehole tap water at central places. Collected specimen samples will be transported in cold boxes to the MRC Keneba laboratory in West Kiang, The Gambia, for laboratory procedures.

### Participants

In total 393 healthy young children, aged 6-23 months, will be identified during child welfare clinics at the health facilities of Jarra West and Kiang East. After informed consent is obtained, children will have to meet the inclusion/exclusion criteria to be enrolled into the study. For inclusion children must be apparently healthy, 6-23 months old, not severely malnourished (z-scores for Height-for-Age, Weight-for-Age, Weight-for-Height > -3 SD), 7 g/dL ≤ Hb < 11 g/dL, free of malaria, resident in the study area, able and willing to comply with the study protocol, have no congenital disorders or chronic disease, not taking regular medication and not participating in another study.

In addition to the main study, ninety children will also be included into a sub-study (measuring iron absorption). The differences in the sub-study inclusion criteria are that eligible children must be aged between 6-8 months and 7 g/dL ≤ Hb < 12 g/dL when the stable isotope is administered at enrolment.

### Study design

This is a proof-of-concept, three-arm, double-blind randomized controlled non-inferiority trial. The three arms are:

A) Supplementation with a MNP containing 12 mg iron daily
B) Supplementation with a MNP containing 12 mg iron/day if hepcidin is below 5.5 ng/mL, or MNP containing 0 mg iron/day if hepcidin is ≥ 5.5 ng/mL, based on a weekly hepcidin screening indicating whether iron can be given for the next 7 days
C) Supplementation with a MNP containing 6 mg iron/day if hepcidin is below 5.5 ng/mL, or MNP containing 0 mg iron/day if hepcidin is ≥ 5.5 ng/mL, based on a weekly hepcidin screening indicating whether iron can be given for the next 7 days

### Recruitment, enrolment and randomisation

**Fig. 1:**
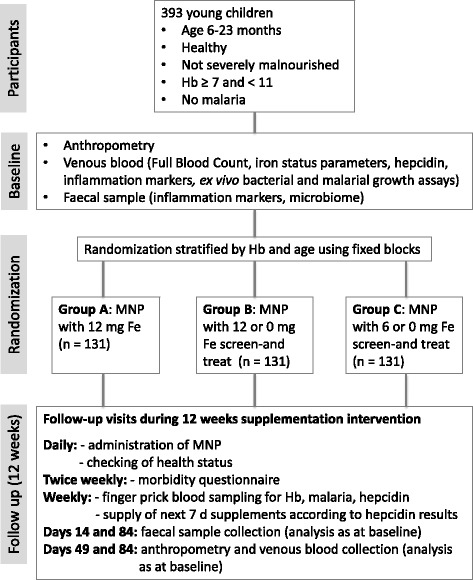
Main study flow chart. Legend: Hb, Haemoglobin; Fe, Iron; MNP, Micronutrient powder

A summary chart of all study procedures is illustrated in Fig. 1. Mothers/Guardians of the young children identified during child welfare clinics at the selected health centres will be visited at home where the study will be clearly explained. Demographic information will be collected once the child’s mother/guardian has signed the informed consent form. If the inclusion criteria are met, the child will be invited for further eligibility screening during which they will be physically examined. Height, weight, head circumference, mid-upper arm circumference and triceps skinfold thickness will be measured and a finger prick blood sample for Hb and rapid diagnostic test (RDT) for malaria will be taken. If Hb is ≥ 7 and < 11 g/dL, and the RDT is negative, a venous blood sample of 5 mL will be collected. The mother will then be asked to collect a stool sample from the child within the next two days. Enrolled children will be randomized to one of the three study arms (equal number in each arm) using a block randomization, balanced by Hb concentration and age, to minimise potential baseline imbalance.

Children with Hb < 7 g/dL will not be enrolled and will be referred to the regional health centre for treatment according to national guidelines. Children with Hb ≥ 11 will not be enrolled as they do not need iron supplementation. Malaria positive children (positive RDT and confirmation by blood film) will not be enrolled and will be treated according to national guidelines.

**Fig. 2:**
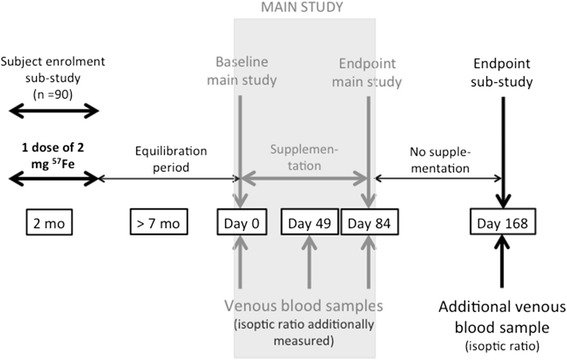
Sub-study flow chart and timing. Legend: mo, month

The 90 children additionally participating in the sub-study will be recruited as described above, but the enrolment day procedure is slightly different (Fig. 2). Children eligible for the sub-study will not have a venous blood sample drawn, but will receive the stable isotope dose on the day of enrolment. They will then be followed up monthly using a morbidity questionnaire until their participation in the main study (earliest seven months after stable isotope administration). This period is to ensure that the stable isotope has homogenously equilibrated with total body iron. On the day of enrolment into the main study, these children will undergo the same procedure as described above for the main study.

Recruitment into the sub-study has started in April 2014 while the first cohort for the main study was recruited in May 2014.

### Follow-up

Field workers will visit all children daily during the 12 weeks supplementation period in order to supervise the MNP administration and to check on the children’s health status. Twice weekly morbidity data (including questions regarding fever, diarrhoea, vomiting, cough, any other illness, appetite and any medication taken and assessment of body temperature) will be captured. If a child is found unwell, the study nurse will check on the child and decide on the appropriate treatment. Each week every child will be screened using a finger prick blood sample (200 μL) to determine their hepcidin and Hb level and their malaria status. Hb and RDT testing will be directly conducted in the field whereas hepcidin analysis will be performed in the laboratory in Keneba. On the following day hepcidin results are used to allocate the MNP for the next seven days for each child. Supervised MNP administration will be conducted by the field workers daily at participant’s homes. During the trial, children found with a positive RDT will be further tested with a blood film and treated according to national guidelines if malaria is confirmed. Any child with a Hb < 7 mg/dL will be excluded and referred to the next health centre for management according to national guidelines.

On Days 14 and 84 another faecal sample and on Days 49 and 84 another venous blood sample (5 mL) will be collected, instead of a finger prick blood sample. All blood samples will be kept in a cool chest after collection and transported to Keneba on ice. An additional blood sample will be collected on Day 168 from the children also participating in the sub-study. Height, weight, head circumference, mid-upper arm circumference and triceps skinfold thickness will be re-measured at Days 49 and 84, as well as Day 168 (for the sub-study children).

### Investigational product

The investigational product to be used in this trial is a MNP (MixMe WHO) produced by the DSM Company and distributed routinely by the United Nations Children’s Fund and the World Food Programme (contains 10 mg of iron). For our study purposes the iron concentration will be altered and we will use products containing 12 mg, 6 mg and 0 mg iron/sachet (daily dose). Each sachet will consist of the micronutrient powder described in Table 1. All sachets will look the same, which will help ensure that field workers, study nurses, participants and the principle investigator are blinded.

**Table 1:** Vitamin and mineral content in a single daily dose of the micronutrient powder (sachet)

Micronutrients	Dose/day
Vitamin A (ug RE)	400
Vitamin D (ug)	5
Vitamin E (mg)	5
Vitamin C (mg)	30
Thiamine (B_1_) (mg)	0.5
Riboflavin (B_2_) (mg)	0.5
Niacin (B_3_) (mg)	6
Pyridoxine (B_6_) (mg)	0.5
Cobalamine (B_12_) (ug)	0.9
Folate (ug)	150
*Iron (encapsulated ferrous fumarate) (mg)*	*12 or 6 or 0*
Zinc (mg)	4.1
Copper (mg)	0.56
Selenium (ug)	17
Iodine (ug)	90

Children additionally participating in the sub-study will receive a single dose of 2 mg iron, in the form of ^57^Fe-enriched ferrous sulphate, at the day of enrolment into the sub-study.

### Laboratory evaluations

#### Blood samples

In the *venous blood samples* collected on Days 0, 49 and 84 the following parameters will be assessed: full haematology panel (using a Medonic M^20M^ GP); ferritin, soluble transferrin receptor (sTfR), serum iron, transferrin, transferrin saturation (TSAT), c-reactive protein (CRP), alpha 1-acid glycoprotein (AGP) (using a fully automated biochemistry analyser Cobas Integra 400 plus); and hepcidin (using the Hepcidin-25 (human) EIA Kit (Bachem) and a Thermo Scientific Multiskan FC Microplate Photometer). Red blood cells (RBCs) will be lysed for measurement of riboflavin status by the erythrocyte glutathione reductase activation coefficient (EGRAC) test.

*Ex vivo* growth of *P. falciparum* will be assessed in washed RBCs using a field-ready 96-well plate method and a basic flow cytometry readout (with the BD Accuri CSampler) in which parasitized RBCs (pRBCs) are identified by DNA dye SYBR green I (Life Technologies). Briefly, cultures will be seeded into RBCs from study participants as rings at 0.5 % initial pRBCs and 1 % haematocrit in triplicate into 96 well plates and maintained for 96 h. Parasite growth rates will be determined by dividing final parasitaemia at 96 h by initial parasitaemia at 0 h [17].

*Ex vivo* growth of sentinel bacteria (*Staphylococcus aureus*, *Staphylococcus epidermidis*, *E. Coli* and *Salmonella* Typhimurium) in participant serum will be assessed by optical density plots confirmed by baseline and end-point colony counts using the cell culture counting facility of a Thermo Scientific Multiskan FC Microplate Photometer [18].

From the Day 0 whole blood samples genotyping for haemoglobinopathies will be done using haemoglobin electrophoresis and DNA will be extracted using the salting out method to look at genetics of iron metabolism. Known genetic risk factors to be assessed include alpha-thalassemia, glucose-6-phosphate dehydrogenase deficiency and sickle traits. Furthermore, putative functional and key tagging variants in iron regulatory and inflammatory pathways will be screened. Much of this will be available from the Illumina Infinium Human Exome Bead Chip (Exome Chip). A specific ‘iron chip’ may be developed.

In addition to all the analyses done with the venous blood samples for the main study (Days 0, 49 and 84), the isotopic ratio will be measured in RBCs of sub-study participants using an Inductively Coupled Plasma Mass Spectrometer (ICP-MS). In the additional blood sample taken in this subgroup of participants on Day 168, isotopic composition, full haematology, ferritin, sTfR, serum iron, transferrin, TSAT, CRP and AGP will be measured as described above.

In the weekly *finger prick blood samples* Hb using a HemoCue 301 and a RDT (SD BIOLINE Malaria Ag P.f, Standard Diagnostics, Inc.) will be directly performed in the field. In case of a positive RDT a blood film will be prepared and read at the laboratory in Keneba. Hepcidin collected into a BD microtainer® will be measured in the laboratory in Keneba as described above for venous blood.

#### Stool samples

Samples at Days 0, 14 and 84 will be collected in a sterile container with a tight, screw top lid that includes an Anaerocult® sachet (Merck, Darmstadt, Germany) to create an anaerobic environment. The samples will be aliquoted and frozen at -20 °C. Calprotectin (fCAL ELISA, Bühlmann Laboratories AG, Schönenbuch, Switzerland) will be assessed using ELISA methods. The gut microbiota composition will be assessed using 16S rRNA analysis and qPCR on enterobacteriaceae and target commensal bacteria.

### Data entry and handling

Field data will be directly entered into handheld devices (Samsung Galaxy Tab 3 SM-T111) using the Cellica Database Software and synced into the database via a direct secure connection over the 3G mobile network. Field measurements/readings will additionally be entered into a separate physical data collection sheet at each measurement station. These data will be entered by the data entry clerks to serve as the second entry. A double entry verification system is executed to detect discrepancies between the two entries. Discrepancies will be sent as queries to the Principal Investigator for resolution.

### Sample size

This is a non-inferiority trial to evaluate efficacy and the primary endpoint is Hb concentration (measured using a Medonic haematology analyser in the laboratory) at day 84. Based on a SD of 1.15 g/dL of the mean Hb value of children at 24 week of age, from a previous trial conducted at Keneba [19], a sample size of 131 participants in each of the three arms is required using a 1-sided α of 2.5 and a Bonferroni correction to adjust for multiple testing. A total sample size of 393 children followed up for 12 weeks, with a drop-out of less than 15 %, will provide 80 % power to establish that:

1. arm B is non-inferior to arm A on the primary endpoint (Hb concentration at Day 84) defined as the upper 98.3 % confidence limit for the difference in mean Hb being not greater than 0.5 g/dL (non-inferiority margin), the smallest value considered to be of public health relevance
2. arm C is non-inferior to arm A at the same level as above
3. arm C is non-inferior to arm B at the same level as above

For the sub-study, the power calculation for iron absorption is based on the data from Fomon et al. [20] in infants. Twenty subjects per group are required to resolve a difference in absorbed iron of 0.080 mg/d between the control and intervention period, based on a SD of 0.13 mg/d (paired t-test). The between group difference that we estimate being able to resolve is 0.12 mg/d absorbed iron (unpaired *t*-test). Lower variability is expected in the calculated k_abs_ values, which are independent from Hb, body weight, and iron status, the main contributors to variability in the calculation of iron absorption. Considering attrition due to the long time span of at least 7 months between recruitment (administration of stable isotope dose) and participation in the main study, 30 subjects per group will be recruited.

### Statistical analysis

The primary haemoglobin outcome (measured using a Medonic haematology analyser in the laboratory) at day 84 will be tested for non-inferiority by performing a per-protocol analysis with a 5 % significance level, adjusted using the Bonferroni correction to account for three main comparisons. We will also repeat the main analysis using the intention-to-treat population as a confirmatory analysis.

Secondary outcome analysis of continuous variables (ferritin, sTfR, transferrin, TSAT, CRP, AGP, calprotectin, *ex-vivo* growth, gut microbiota, number of weeks supplemented) will be done using mixed-effect models with individuals treated as a random effect, and secondary outcome analysis of categorical variables (anaemia, ID and IDA) will be modelled using mixed-effect logistic regression. Interactions between study arm and time in the study will be investigated.

Morbidity data will be analysed using mixed-effect models controlled for possible confounders.

Exploratory analysis will be performed using serum iron.

All data will be analysed using Stata v12.1 (StataCorp, LP, USE, http://www.stata.com) and statistical significance will be defined as *p* < 0.05, unless stated otherwise.

In the sub-study, the linear regression of log^57^A^t^ (^57^A^t^ = abundance at time t) against time will be calculated for each subject belonging to the three different groups for the period of Day 0 to Day 84 (with a midpoint sample at day 49). The slope (k_abs_) of the regression reflects the absorption of iron per unit of time. The difference in the slopes between the groups will be equivalent to the average iron absorption over time in each group (k_abs_ in groups A, B, and C, respectively).

The assumption of homogenous labelling will be assessed by testing the linearity of the slope between Days 0, 49 and 84. In each subject, k_abs,i_ will be calculated from the slope of the isotopic ratio decrease over the intervention period. k_abs,c_ will also be calculated for the control period (Day 84 and 168). k_abs_ will be modelled using linear mixed models with time (intervention and control period) and treatment as fixed factors and subjects ID as random factors. The time treatment interaction on k_abs_ will be assessed. A similar but separate analysis will be conducted on the total amount of iron absorbed, which will be estimated from the k_abs_ value in each subject, and the group and time effect will be modelled with linear mixed models. The models will be compared to the standard treatment of supplementation with 12 mg iron/day universal.

## Discussion

Prevalence of anaemia is > 50 % and remains a major public health problem in young children in The Gambia. Combating anaemia due to iron deficiency is a challenge due to the potential negative side effects of iron when given to people with infections, as is common in The Gambia. The present randomised controlled trial of screen-and-treat aims to minimise unnecessary exposure to iron. This will decrease the potential adverse effects of giving iron at a time when the body can’t utilise it and will allow maximising the absorption and utilisation of iron when it is most needed. If a screen-and-treat approach using a hepcidin cut-off to define ‘ready and safe to give iron’ is non-inferior to universal supplementation, then this approach could be used in every health centre. We hypothesise that the development of a point of care test, which would immediately tell the nurse/physician if the person is ready to receive iron, would make iron administration more targeted and safer.

## Abbreviations

AGP: Alpha 1-acid glycoprotein
CRP: C-reactive protein
Hb: Haemoglobin
ID: Iron deficiency
IDA: Iron deficiency anaemia
MNP: Micronutrient powders
MRC: Medical Research Council
RBC: Red blood cell
RDT: Rapid diagnostic test
SD: Standard deviation
sTfR: soluble transferrin receptor
TSAT: transferrin saturation
WHO: World Health Organization
